# Supplementary material for: Attachment-Based Compassion Therapy for Reducing Anxiety and Depression in Fibromyalgia
Source: Int J Environ Res Public Health. 2022 Jul 2;19(13):8152. doi: 10.3390/ijerph19138152 (PMC9265807; doi:10.3390/ijerph19138152)
Supplement: Supplementary file 1 [file ijerph-19-08152-s001.zip › ijerph-1723734-supplementary.pdf]

SUPPLEMENTARY MATERIAL

Supplementary Table S1. The ABCT-16

**Session 1**	
**Theory**	The workings of our brain
**Formal practice**	Compassionate breathing and compassionate body scan
**Informal practice**	3-min compassionate practice
**Tasks**	Compassionate breathing and body scan<br>3-min compassionate practice<br>Diary of self-compassion and practice
**Session 2**	
**Theory**	The reality of suffering: primary and secondary suffering<br>What is and isn’t compassion?
**Formal practice**	Compassionate coping with difficulties
**Informal practice**	Savoring and giving thanks
**Tasks**	Compassionate breathing and body scan<br>Compassionate coping with difficulties<br>3-min compassionate practice<br>Diary of self-compassion and practice
**Session 3**	
**Theory**	Compassion and mindfulness<br>Self-esteem and compassion
**Formal practice**	The figure of affect: connecting with basic affection<br>Developing a safe place
**Informal practice**	The object that joins us to the world
**Tasks**	Compassionate breathing and body scan<br>Figure of affect (formal and informal)<br>Safe place (formal and informal)<br>3-min compassionate practice
**Session 4**	
**Theory**	Fear of compassion
**Formal practice**	The compassionate action<br>Identifying the figure of secure attachment

**Informal practice**	What are we good at?
**Tasks**	Compassionate breathing and body scan<br>Figure of affect (formal and informal)<br>Safe place (formal and informal)<br>The compassionate action (formal and informal)<br>3-min compassionate practice
**Session 5**	
**Theory**	How compassion acts
**Formal practice**	Developing the figure of secure attachment
**Informal practice**	-
**Tasks**	Compassionate breathing and body scan<br>Develop and stabilize the figure of secure attachment
**Session 6**	
**Theory**	Effectiveness of compassion<br>Self-criticism
**Formal practice**	Replacing the self-critical voice with a compassionate voice
**Informal practice**	Writing a letter to a compassionate friend
**Tasks**	Compassionate breathing and body scan<br>Diary of Self-Critical Voice<br>Replace the self-critical voice with the compassionate voice once a day<br>Writing a letter to a compassionate friend
**Session 7**	
**Theory**	Attachment styles<br>Importance of these styles in everyday life
**Formal practice**	Becoming aware of our attachment style
**Informal practice**	-
**Tasks**	Compassionate breathing and body scan<br>Working on attachment style<br>3-min compassionate practice
**Session 8**	
**Theory**	-
**Formal practice**	Ability to receive affection: friends, indifferent people, enemies

**Informal practice**	Letter to your parents
**Tasks**	Compassionate breathing and body scan<br>Working on the ability to receive affection: friends, indifferent people<br>Write the letter to your parents
**Evaluation session**	
Short review of the contents of the program<br>Self-esteem exercise: Participants are given a sheet of paper with a big circle drawn on it. They are asked to write their name inside. After that, using colored pens they must write their virtues. 10 minutes later they are given another sheet of paper with a list of positive adjectives, so they can find more positive aspects of themselves to include in the circle. Finally, they are instructed to decorate the external part of the circle and to include a positive sentence that may help them in tough times. They are asked to take a photo of the drawing with their cell phones and to put the original somewhere easy to find or see, to revise it when they do not feel OK.<br>Questionnaires	
**Session 9**	
**Theory**	Importance of affection towards ourselves and others
**Formal practice**	Showing affection to friends and indifferent people<br>Showing affection to ourselves
**Informal practice**	The greatest display of affection (in general and from our parents)
**Tasks**	Compassionate breathing and body scan<br>Working on attachment style<br>Working on receiving affection from friends and indifferent people<br>3-min compassionate practice
**Session 10**	
**Theory**	-
**Formal practice**	Reconciliation with our parents<br>Three positive aspects and three negative aspects of our parents
**Informal practice**	The illusion of labels
**Tasks**	Compassionate breathing and body scan

	Working on attachment style<br>Working on receiving affection from friends and indifferent people<br>3-min compassionate practice<br>The illusion of labels
**Session 11**	
**Theory**	Guilt and the importance of forgiveness
**Formal practice**	Forgiving yourself<br>Asking others for forgiveness
**Informal practice**	Interdependence
**Tasks**	Asking others for forgiveness<br>Interdependence
**Session 12**	
**Theory**	-
**Formal practice**	Forgiving others and showing compassion to enemies<br>Showing forgiveness for the hurt caused by loved ones (only for people with this experience)<br>Recapitulation
**Informal practice**	Compassion in everyday life
**Tasks**	Forgiving others and showing compassion<br>Informal compassion
**Session 13**	
**Theory**	Working in three periods<br>Envy<br>Usefulness of being our attachment figure
**Formal practice**	Working with envy<br>Becoming our own attachment figure
**Informal practice**	-
**Tasks**	Asking others for forgiveness<br>Forgiving others and showing compassion<br>Interdependence<br>Informal compassion
**Session 14**	

**Theory**	Difficult people
**Formal practice**	Handling difficult relationships
**Informal practice**	Not taking anything personally<br>Looking others in the eye and connecting with their suffering
**Tasks**	Asking others for forgiveness<br>Forgiving others and showing compassion<br>Interdependence<br>Informal compassion
**Session 15**	
**Theory**	Equanimity
**Formal practice**	Equanimity I: We are all equal.<br>Equanimity II: The illusion of categories
**Informal practice**	Our values and their relation with compassion
**Tasks**	Asking others for forgiveness<br>Forgiving others and showing compassion<br>Interdependence<br>Informal compassion
**Session 16**	
**Theory**	How to keep up the practice of compassion for a lifetime
**Formal practice**	Equanimity II: Showing the world the gratitude we have not been able to give back
**Informal practice**	The cosmic attachment figure<br>The tantric embrace
